# Essentials of Chemistry, Inorganic and Organic

**Published:** 1878-12

**Authors:** 


ESSENTIALS OF CHEMISTRY, inorganic and organic, for the use of students in medicine. By R. A. Witthaus, A.M., M.D., Professor of Chemistry in the Medical Department of the University of Vermont, etc., etc. New York: Wm. Wood & Co. 1879.

This is a very small book of about 250 pages, and seems to be intended for convenient reference and study, as it can be carried readily in the pocket.

We are in receipt of "The Physician's Visiting List for 1879," which is the "twenty-eighth year of its publication."—Philadelphia: Lindslay & Blakeston. Sold by all booksellers and druggists.

Also, "The Physician's Pocket Day-Book, by C. Henri Leonard, M.A., M.D." Price $1.00—Detroit, Mich.

These are conveniently arranged for the use of practicing physicians, to be carried regularly in the pocket. The former we have been using regularly for some years, and find it useful for keeping medical accounts and calls to be made.

The latter being thinner and also conveniently arranged for noting calls made and to be made, will be found equally useful for the use of the general practitioner.

